# Challenges on Radical Health Redesign to Reconfigure the Level of e-Health Adoption in EU Countries

**DOI:** 10.3389/fpubh.2021.728287

**Published:** 2021-07-20

**Authors:** Magda Mihaela Luca, Lavinia Mustea, Alexandra Taran, Petru Stefea, Sorana Vatavu

**Affiliations:** ^1^Department of Dentistry, Victor Babeş University of Medicine and Pharmacy, Timisoara, Romania; ^2^Department of Finance, West University of Timisoara, Timisoara, Romania; ^3^Department of Management, West University of Timisoara, Timisoara, Romania

**Keywords:** e-health adoption, relative distances method, composite index, digitalization, EU

## Abstract

The recent worldwide COVID-19 pandemic has highlighted the importance of a performant public sector in terms of health. To achieve greater use and efficiency of health information and communication technology (ICT), the whole community of European states needs a model to develop a common strategy to support the implementation of e-health and reduce decision-making difficulties. Our research suggests such a model, starting from the level of adoption to the implementation of e-health and points out the existing disparities in the European countries regarding the difficulties of adopting e-health. We draw a composite index to assess the inequalities present in the quality of life, the public health system, and the adoption of e-health. Furthermore, to return to a hierarchy of European countries, the relative distance method (RDM) is applied by combining various classification criteria. The results identify the European countries with the highest levels of adoption (Denmark, Estonia, Spain, Sweden, Finland, and the United Kingdom), where e-health is routine, and the countries with the lowest levels of adoption (Greece, Lithuania, Luxembourg, Malta, Romania, and Slovakia), where e-health is not widespread. These results reveal critical implications in identifying solutions to reduce the gaps between countries, identifying public policies to support the adoption of e-health, and reducing difficulties in decision-making.

## Introduction

The government plays the primary role in ensuring the well-being of the people and also has the responsibility to ensure the health of the people. On the other hand, ministries of health must provide policies that contain the most effective and appropriate choice in creating and delivering a high-performance health system. However, the society of today is categorized as a knowledge society; hence, supremacy and authority no longer depend entirely and are no longer focused only on government. Informed citizens, the organizations involved, agencies, and expert bodies have an increasingly significant role to play in the society and whose opinions weigh heavily in decision-making. The health system can only meet all our needs if there is a common goal of government and society, for which there must be coherence between the decisions taken.

In the present crisis time caused by the COVID-19 pandemic, of fiscal and demographic crises, when economic growth is uncertain, it becomes apparent not many nations will be shaped by the health of their population. E-health plays a crucial role in achieving a European community that is predefined by the existence of a healthcare system characterized by the following key terms: quality, increased safety, and high efficiency. Within the e-health community, close communication is needed to form and clarify a precise path leading to fulfilling the common vision of the main actors: government, private industry leaders, and academia (1).

Although e-health is proven to improve healthcare quality, the adoption of e-health dimensions, such as electronic health record (EHR), health information exchange (HIE), telemedicine, and personal health record (PHR), is either low or unused in European countries. This is partly due to factors that affect the perceptions of citizens, the barriers and challenges that health professionals face, and how professionals come to accept this new system and then make it available to the infrastructure. Although the components, such as EHR, HIE, telemedicine, and PHR are the essential tools to improve the safety and quality of healthcare, the medical field still faces some shortcomings due to high costs and difficulties in their implementation, despite the clear benefits that it can bring in the integration of information and communication technology (ICT) in the health area. According to Sapirie (2), there is a large gap between planning the adoption of the new technology and in the sustainable implementation of such technology to obtain future strategic or expected benefits.

Good health technology management is a prerequisite for increasing the efficiency of health services. The need to do more with less is essential, as the health sector faces increasing demands while receiving static or declining resources.

The use of ICT in the medical field is of significant importance, especially in the contemporary society, facing a health crisis, the impact of the pandemic requiring transfer to the online environment, and the use of ICT. Thus, ICT brings important benefits in improving the quality and the delivery of medical services, reducing costs, increasing revenues, increasing patient safety, reducing waiting times, and creating greater patient engagement during its care (3).

In the recent years, e-health has significantly expanded in Europe. In our paper, we endorse the definition of e-health as the use of ICT in health services, products and processes combined and adapted to organizational changes in health systems, and new concepts to improve the health of the citizens, and at the same time, providing productivity in healthcare. Simultaneously, the health system will benefit from improvement in the economic area and its social value.

In the recent year, health has not only shaped the modern state and its social institutions in Europe in, but it has also fueled social movements, defined the rights of the citizens, and contributed to building the modern self-concept and aspirations (4).

Once again, the worldwide crisis (COVID-19 pandemic) generated an economic downturn and demographic change and highlighted the importance of the health system of a country. More precisely, the future of a country is related to the health of its population, and nowadays, the rapid development of the health system is crucial. The level of economic diversification in countries is also important as it might reduce the lag in the development of different sectors. As the health system currently needs a strong support, investments are necessary and a greater institutional quality might lead to an improvement in the determinants of growth process, reducing the lag and development differences across sectors (5).

Even though health is essential for the well-being of individuals and for the progress of the society, political discussions acknowledge it as the provision of health services. Many countries, especially those with low incomes, do not yet have an effective health system, while others face difficulties with the basic mechanisms of e-health governance, such as ensuring financial protection for users. Richer countries must also remain vigilant and also address e-health inequalities. These concerns are and will remain challenges to the public sector.

Result-oriented management and leadership and patient-centered care processes are two of the concepts related to e-health. Furthermore, the literature reveals that IT employment in the health systems will produce revolutionary results (6). Moreover, special attention is accorded to the IT impact on the doctor–patient relationship. If health systems are seen as ways to organize access to specialized knowledge, then it is essential to have an access to knowledge and in organizing it (7).

In this case, e-health can provide enormous health benefits and help in the management of health systems in the future. However, the benefits offered are not automatic but require governance on three issues: monetary cost, policy, and utility.

The general opinion is that the functions of an information system at a national level are broad. Thus, at the European level, the infrastructure, systems engineering tools, and associated techniques for the design, regulation, and improvement of health processes must expand and align with the 2004 “European e-Health Action Plan” (8).

Popovic et al. (9) stated that the rapid advances in ICT and the growing number of intelligent devices allow the transfer of health and healthcare resources by electronic means. E-health is linked to the Internet, which provides a new environment for disseminating healthcare and interaction and collaboration between institutions, health professionals, healthcare providers, and the public. In developing countries, e-health is particularly important due to shortage of doctors and nurses. Although most doctors in developing countries recognize the benefits of e-health, adoption is low.

Applications of e-health technology are essential tools that enhance healthcare provision quality in hospitals in developed and developing countries. Regardless of its benefits, the literature reveals that e-health adoption in developing countries is still reduced. Some of the reasons are due to barriers, such as the resilience of health professionals, poor infrastructure, and reduced technical expertise (10). It was found that performance expectation, effort expectation, social influence, and personal innovation significantly impacted the behavioral intention to use e-health, while facilitation conditions did not have any significant effect (11).

Murray et al. (12) explored the experiences from the perspective of the implementers—senior managers and other employees responsible for implementing e-health initiatives—and evaluating factors that promote or inhibit the implementation, incorporation, and successful integration of e-health initiatives.

More recently, Devlin et al. (13) considered normalization process theory (NPT) to analyze data on the “Dallas” program in the United Kingdom. The results identified five key challenges: (i) the challenge of establishing and maintaining heterogeneous, large multi-agency partnerships to provide new healthcare models; (ii) resistance to barriers and continuous attention to changes in external environments; (iii) the inherent tension between innovative co-design and timely and scaled delivery; (iv) the effects of branding and marketing problems in consumer care environments; and (v) challenging interoperability and information governance when proprietary business models are dominant.

In 2004, the European Commission launched an initiative to adopt the first e-Health Action Plan. Thus, Lang and Mertes (14) analyzed the implementation of 12 e-health policies and explained the variation in e-health tools (applications) among 24 EU Member States.

Melchiorre et al. (15) analyzed 24 European countries based on the project, “ICARE4EU.” The study highlights certain aspects, which are found to be benefits (integration/management) or barriers (cost-effectiveness and quality of care/life) for e-health adoption. Furthermore, the findings are subsequently linked to the “10 es” in e-health: “Efficiency, Enhancing, Evidence-based, Empowerment, Encouragement, Education, Enabling, Extending, Ethics, and Equity.” Thus, the results can represent new support objectives for implementing e-health technologies in integrated care across Europe. Peek et al. (16) found that the use of technologies by the elderly was complex, dynamic, and personal. The results indicate that periods of both stability and change occur naturally. Moreover, the framework of Dynamics in Technology Use by Seniors (DITUS), followed in the Netherlands, can help understand the stability and instability of using technology and the development and implementation of sustainable technological solutions. Therefore, the results reveal that a core of six correlated factors was closely related to the frequency of technology use: emotional attachment, compatibility of needs, cues to use, the competence of use, resource inputs, and support. In addition, disruptive forces, such as social influences, competition with alternative means, and changes in personal needs, could induce changes by affecting these six factors.

Furthermore, the study conducted by Arena et al. (17) empirically examined Italian public hospitals and used a combined measure of innovation based on different e-health solutions. The results show that women managers encourage the implementation of innovative strategies and facilitate the adoption of e-health. The results also show that gender similarity increases the rivalry between the top management team and the line managers, thus limiting the adoption of e-health solutions. Johansson et al. (18) investigated the experience of digital primary health system (DPHS) using written dialogues in Sweden, highlighting that the examination of patient experiences can support decision-making in expanding the digital healthcare. The main results reveal that patients felt well-prepared and experienced in various aspects; some patients would recommend digital primary health care (DPHC) to others, and a notable reason for satisfaction was available. However, patients expressed some uncertainty about the ability of a doctor to assess the correct care needs. The results of the authors can be a knowledge base that will be useful for other areas and for countries that encourage implementing digital health services in primary healthcare systems.

Hantrais et al. (19) conducted a study through which they gathered evidence from different areas about the impact of COVID-19 in digital societies and identified policy responses in this context. The authors showed how the pandemic produced changes not only in data collection techniques and in the practices of disseminating official statistics, but also in the way in which the seemingly insurmountable obstacles to the implementation of health treatments managed to be largely overcome. The results obtained by the authors confirm that the ethics of emotional intelligence has become a significant concern for government legislation at the national and international levels.

Our study makes three contributions. First, it identifies the levers that could create the momentum for change in e-health adoption, setting out the preconditions and benefits for different groups of countries. Secondly, the inequalities present in the quality of life and the public health system, respectively, in the level of e-health adoption are assessed in a multicriteria manner, finally implying a hierarchy of countries. Third, this research also takes into account, the distinction of different research topics, which reveals that no significant progress has been made in the recent years. Finally, in the context of continued digitalization, this research concludes and affirms the need for public policies, structural and decision-making reforms, and joint action plans through which EU countries can reach a significant threshold on all matters, which essentially means adopting e-health. European Union countries need to consider common approaches in all aspects involved in the adoption and implementation of e-health, as evidenced by the uniform and interoperable approach to vaccination or to the development of effective applications for monitoring and warning of COVID-19 infection.

The rest of the paper is structured as follows: section Materials and Methods depicts the data and methodologies used in this paper, section Results reveals the obtained results, section Discussion discusses and presents robustness and empirical analysis, and section Conclusion provides the conclusion.

## Materials and Methods

Considering that public health is a prerequisite for continuous development, the need for sustainable improvement and constant investment in e-health adoption is stated, which would lead to a sustainable increase in the quality of life and in the efficiency of the public health system.

Thus, a wide range of methodologies have been applied, which are as follows:

- Statistical analysis focusing on disparities in individual health (measured by the quality of life), EU28. Statistical analysis by cluster technique uses KNIME software and the K-means clustering algorithm.
- Statistical analysis focused on disparities in the quality of the public health system, EU28;
- Statistical analysis focused on disparities in e-health adoption, based on the ranking of the country, EU28;
- Correlation econometric analysis— finding the intensity of possible links between e-health adoption level and country rank (depending on the public health system and quality of life)—the analysis used to identify a common policy orientation to be applied to all Member States of EU, which envisages the implementation of the information and communication technologies (ICT) on health, to increase the quality of life, the public health system, and significantly reduces the existing disparities among the EU Member States;
- Level of implementation and use of compound indices, such as EHR, HIE, telemedicine, and PHR—finding the level at which each country is and also highlight the subdimensions and functionalities that are already used or in use, in order to draw the defining lines which can lead to the adoption at a more significant level of these functionalities, respectively of the others.

To capture particularities, many variables were taken into account, made use of the relative distances method to combine different criteria, and to obtain the hierarchy.

We followed the methodology used by the report of the European Commission (20, 21) and the study by Lobont et al. (8), namely the calculation of composite indices. We used PCR to compute our indices, considering that this methodology allows us to transform our more extensive set of variables (some of them correlated) into an uncorrelated smaller set of variables that captures most of the variations from the original set. The RDM allows us to classify and rank countries, indicating the countries that managed to implement and successfully apply the specific e-health functionalities. This ranking analysis also allowed us to focus on disparities in the quality of the public health system and disparities in e-health adoption. In addition to other studies which only employed the RDM method [e.g., (22, 23)], we also included a regression analysis to emphasize the factors which mostly influence the quality of life (or a proxy for individual health), and on the efficiency of the public health system. Employing the KNIME software is also an advantage compared to previous studies (even to the latest, similar research undertaken by 8), allowing us to perform a cluster analysis which defined the groups based on the similarities observed among the countries.

The relative distance method helps in the transformation of the initial value into relative distance compared to the most performing value related to each criterion (24, 25). In this section, the RDM allows the observation of the relative distance of each country and that of the country with the highest level at the European level. The relative distance between the best-performing country [according to criterion (j)] and the rest of the countries is measured by the following ratio:

$$\begin{array}{l} \frac{Xij}{Xmaxj} \end{array}$$

Moreover, the relative distances of each classification criterion (j) are calculated. At the same time, as a simple geometric mean of the previous results, the average of the relative distances of each country [for all criteria (j)] is calculated:

(1) $$\begin{array}{r} Di=\sqrt[{m}]{\underset{j=1}{\overset{m}{\prod }}\frac{Xij}{Xmaxj}} \end{array}$$

where, m = the number of criteria used.

Therefore, the country (i) can be classified according to the decreasing average relative distance (Di).

Only through a common basis, we can compare disparities in a country with those in other countries. In order to satisfy the method of relative distances, the average value at the European level will replace the performance recorded by the best country for criterion, j (Xmax j); therefore, the following formula for multi-criteria, provides the result for j, which is the average distance for each country:

(2) $$\begin{array}{r} Di=\sqrt[{m}]{\underset{j=1}{\overset{m}{\prod }}\frac{\text{Xij}}{\bar{\text{Xj}}}} \end{array}$$

The approach taken is the way to reach a firm and the fixed place of each country compared to the European average, making possible comparisons at different levels (both at the European and national levels). For measuring the disparities, economic criteria are employed, which include synthetic indicators that we consider relevant to our analysis and comparable in space. Based on the objectives of this paper and the available databases, only the criteria were selected to reflect the individual health and the quality of the public health system. Also, Jeremic et al. (22) and Seke et al. (23) selected and used some of these indicators in their analysis. The data comes from the European Union databases, such as the WHO, Eurostat, the World Bank (WB), and Statista. To perform the classification of the quality of life (the individual health) in the European countries, we made use of the Physical Quality of Life Index (PQLI) (data source: Eurostat and WB; see Supplementary Table 1).

Considering the quality of the public health system and to classify the European countries, we use the Public Health criteria (data source: Eurostat, European Commission, and Statista; see Supplementary Table 2).

Regarding the adoption of e-health, Lupiáñez-Villanueva et al. (21) conducted a survey funded by the European Commission, a survey of general practitioners (GPs) from 31 countries (EU28 + Iceland, Norway, and Turkey) to measure and explain the levels of availability and the use (adoption) of e-health applications and services. A random sample of 9.196 family physicians was interviewed, and data were processed using sophisticated multivariate statistical techniques. The survey was conducted between January 2018 and June 2018. In the 28 EU countries analyzed, a final sample of 5,793 GPs was randomly selected, with an overall sampling error of ± 1.30%. Univariate and multivariate statistical analyzes were performed to analyze the survey data. Each composite indicator consists of two to five subdimensions, which group the functionalities into broader categories. The grouping of functionalities into subdimensions followed the same approach used in the 2013 study by “Benchmarking Deployment of e-Health among General Practitioners” (20).

The EHR criteria is the system used by healthcare professionals (both physicians and nurses) to enter, store, view, and manage patient health and manage information and data (Supplementary Table 3).

The HIE is the process that includes the electronic transfer/sharing/allowing access to information and data on patient health (see Supplementary Table 4).

Telemedicine—TeleHealth—represents the use of technological platforms based on the broadband for the distribution of distant services, medical training, and health education (Supplementary Table 5).

Personal health record (PHR) reflects an electronic system that allows patients to have secure access and proper management of personal health information (Supplementary Table 6).

The analysis carried out in the report of the European Commission also includes the classification of EU Member States, based on the calculation of the composite index for each dimension and the global e-health adoption index for each European country using the Factor Analysis (FA) method. In our study, we considered comparing the global e-health adoption index developed using FA with the composite index that we constructed using the RDM. The coefficient of determination, R2 on the correlation analysis of the two composite indices reveals that the indices are practically interchangeable (a value of 0.9767).

The first part of the study proposes the classification of European countries according to the quality of life. Besides, many indicators have been taken into account to create the composite index of healthy living quality (Di), but only those such as life expectancy at birth, and healthy life years of women and healthy life years of men provide a different image in terms of the best performance of the indicators. Therefore, of all the indicators taken into account, the highest possible values indicate the best performance of the analyzed indicator of the country. The results obtained are presented in the section 3 and were used to classify the European countries according to the quality of life. The relationship applied to calculate the average of the relative distances is as follows:

(3) $$\begin{array}{r} Di=\sqrt[{7}]{\frac{Xisvn}{\bar{X}isvn}\text{*}\frac{Xifvs}{\bar{X}ifvs}\text{*}\frac{Xibvs}{\bar{X}ibvs}\text{*}\frac{\bar{X}ibps}{Xibps}\text{*}\frac{\bar{X}iamns}{Xiamns}\text{*}\frac{\bar{X}imir}{Ximir}\text{*}\frac{\bar{X}irm}{Xirm}} \end{array}$$

where, Di = composite index of disparities (multicriteria distance compared to the EU28 average for the country “i”);

Xi svn, Xifvs, Xi bvs, Xi bps, Xi amns, Xi mir, and Xi rm = the value corresponding to each country “i” for life expectancy at birth, healthy life years of women, healthy life years of men, people with long-term illness or health problems, unsatisfactory healthcare, and infant mortality

$\bar{\text{X}}$i svn, $\bar{\text{X}}$i fvs, $\bar{\text{X}}$i bvs, $\bar{\text{X}}$i bps, $\bar{\text{X}}$i amns, $\bar{\text{X}}$i mir, and $\bar{\text{X}}$i rm = European average value life expectancy at birth, healthy life years of women healthy life years of men, people with long-term illness or health problems, unsatisfactory health care, infant mortality rate in the country, and mortality rate per month.

## Results

**Figure 1:**
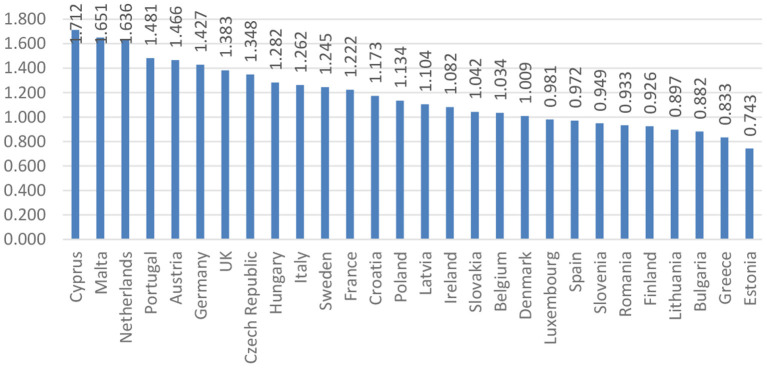
Ranking of the EU countries based on the composite index of disparities of individual health.

A starting point in the econometrical analysis regards the values of disparities (both average and individual), and the fact that they can reflect two situations: a favorable one (when the values are above 1) and an unfavorable one (when the values are below 1). After the actual achievement of the composite index of disparities among individual health, the resulting values formed the basis for the order of the EU Member States. Figure 1 presents the ranking of the EU Member States according to the values obtained.

Figure 1 highlights the ranking of countries in terms of individual health. Thus, countries such as Cyprus, Malta, and the Netherlands are among the countries considered to be the “healthiest” as a result of initiatives taken to provide relevant information to support policy makers and analysts in the development of health systems, including implementation of healthcare reform programs.

Moreover, at the end of the ranking, Greece and Estonia record the lowest values among the countries analyzed. In the case of Greece, the values recorded are due to an aging population, which leads to an increase in long-term health care needs, while there are fewer working-age people to meet these needs. Therefore, the creation of an efficient network of primary care services is one of the most urgent priorities in Greece, in order to be able to respond effectively to the needs of the population. In Estonia, these values are justified by unmet health needs, which are among the highest in the EU, affecting people of all income groups. Other EU countries can provide a source of inspiration for strengthening primary care, but there is probably an issue called “no one fits everyone,” and different models of primary care are likely to coexist and continue to evolve for each country.

The study also aims to regress the composite index of individual health, as a dependent variable, concerning the criteria of the quality of life, as an independent variable. In this sense, from the results obtained from the construction of regression function (linear model), the most significant criteria, such as life expectancy at birth (svn), unsatisfactory care (amns), infant mortality rate (mir), and mortality rate (rm) were selected based on the regression coefficients presented in Table 1.

**Table 1:** Coefficients of determination between the composite index of individual health and the variables of quality of life.

**Independent:**	**svn**	**fvs**	**bvs**	**bps**	**amns**	**mir**	**rm**
Coefficients	0.049^***^	0.007	0.014	0.001	−0.075^***^	−0.068^*^	−0.639^***^
t-Stat	(3.097)	0.840	1.558	0.208	−3.896	−1.852	−3.803
R-Square	0.2695	0.0264	0.0854	0.0016	0.3687	0.1166	0.3575
*F*-test	9.596^***^	0.706	2.428	0.043	15.185^***^	3.432^*^	14.467^***^

^***^, ^**^, ^*^ - significant at 1%, 5%, respectively 10% level.

The resulting coefficients indicate that the most significant variable in determining the quality of life is rm, followed amns, mir, and svn. According to the analyzed statistics, countries such as Cyprus, Malta, and the Netherlands have values reported as the lowest in terms of unsatisfactory healthcare, with a percentage of <0.05% in both the total population and mortality rate. Simultaneously, the recording of such values may be due to the success of the primary care reforms proposed by these countries, a success that probably depended on financial resources, support for innovative ways of efficient service delivery, and effective coordination of different primary care units.

On the other side of the ranking are the countries, such as Greece and Estonia with the highest values related to the variable mortality rate. The decrease in mortality can be mainly attributed to the reduction of important risk factors, such as smoking, especially in men, and to the improvement of the quality of healthcare. Another significant variable is unsatisfactory healthcare, with 9.10% (Greece) and 7.30% (Estonia). In Estonia, high levels of unmet needs can be caused by waiting times for specialist outpatient care, day surgery, and inpatient care. Greeks have difficulty in accessing doctors or a health center not only due to the cost and in the distance to the office of the doctor and waiting for a meeting, but also due to the consultation of a doctor.

Next, through the statistical technique of cluster analysis, it was possible to group and intuitively visualize the discrepancies among European countries. Cluster analysis aims to group the observations to reduce the distance between the observations within the group.

Thus, with the performance of the K-means algorithm, we categorized the data set into k-distinct predefined subgroups (clusters) which do not overlap, so that each data point belongs to a single cluster. This analysis was made possible through the analytical platform, KNIME, the software that offers the possibility to create scientific data. Intuitively and openly, it continuously integrates new developments. The KNIME makes it possible to understand data by designing workflows and reusable components accessible to all.

**Figure 2:**
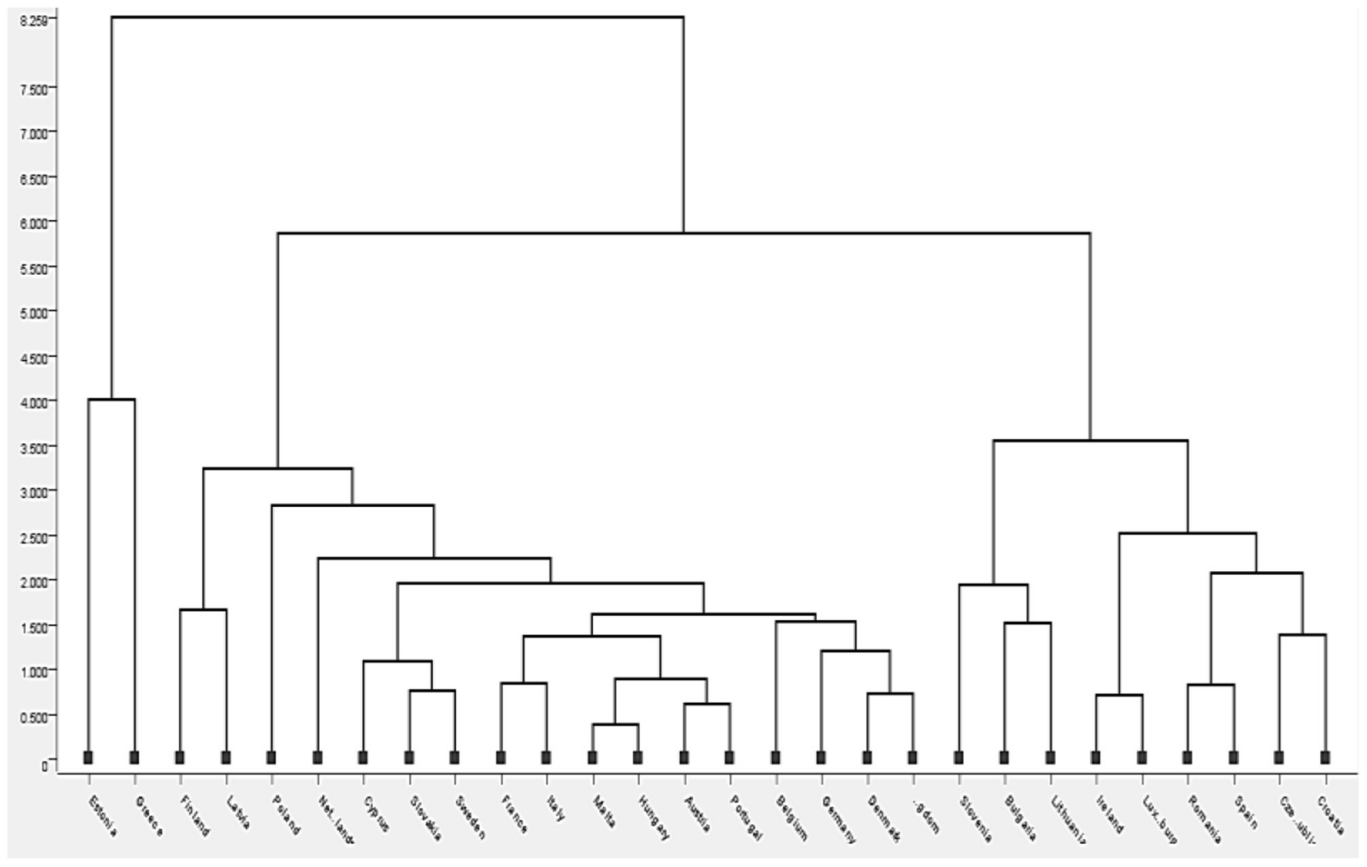
K-means clustering Dendrogram.

This model was implemented in the 28 countries considered in this study and taking into account, the four variables that were the most significant in the previous analysis (Table 1: rm, amns, mir, and svn). Figure 2 presents the clusters obtained through the KNIME analytical platform, using the K-means clustering algorithm. The intuitive distance among countries and/or groups formed is highlighted in the diagram, using a scale from 0 to about 8.5. We can see that the relative distance among the clusters is greater as we move toward the Dendrogram's maximum scale (upwards). Also, the significant distance among the groups means a big difference between them.

**Figure 3:**
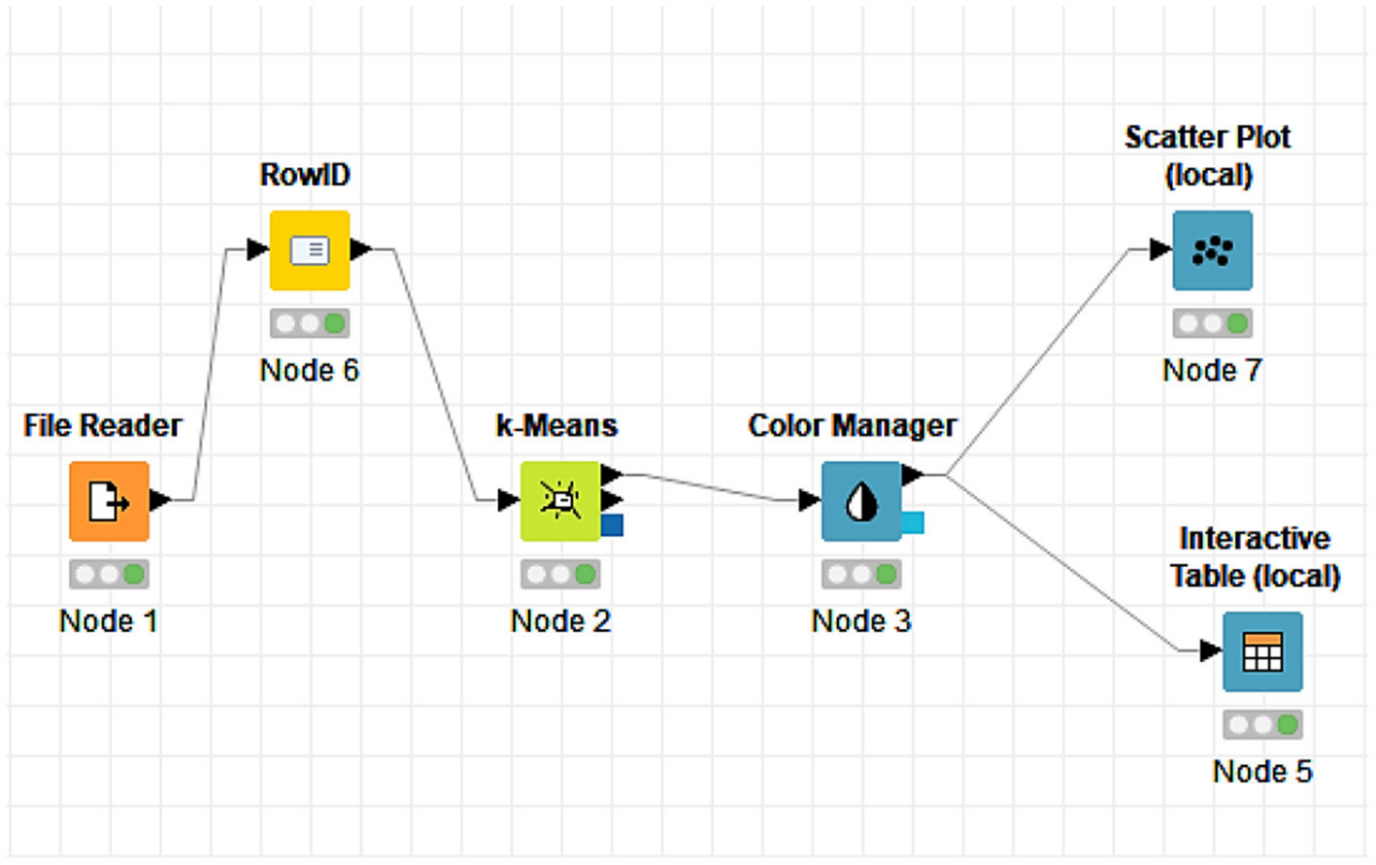
KNIME elements.

**Figure 4:**
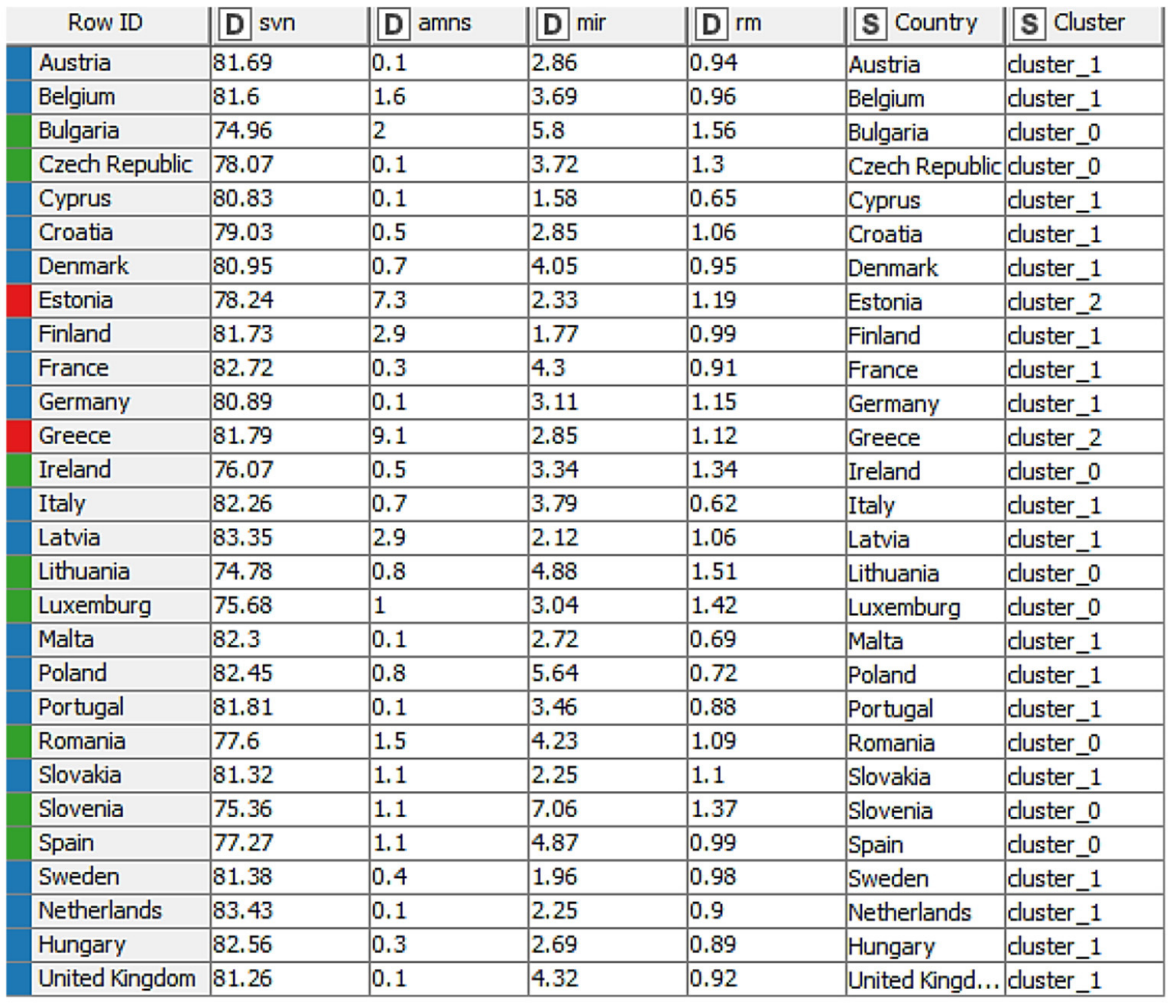
Distributions of countries into groups.

Before moving on to the intuitive specification of the groups formed through the dendrogram, we proposed another way of establishing them, namely the design in the KNIME software with a flow that effectively leads us through several elements selected and connected to the grouping of countries depending on the same data taken into account when creating the dendrogram (Table 1: rm, amns, mir, and svn). Cluster analysis was applied to define groups and to observe the similarity among the countries within each group, to identify the similarities that characterize the different countries and with which they can be grouped in the same cluster. At the same time, Figure 3 depicts the chosen elements, while Figure 4 depicts the results obtained after their connection.

Each element has a decisive role in obtaining the final result, and the K-means algorithm plays an essential role in our analysis. After connecting the elements, the interactive table allowed us to view the distribution of countries and the related groups (Figure 4).

The flow and the chosen elements lead the analysis toward the intuitive formation of three clusters of countries, as presented in Table 2.

**Table 2:** Countries grouped by cluster.

**Cluster 0**	**Cluster 1**		**Cluster 2**
Bulgaria	Austria	Latvia	Estonia
Czech Republic	Belgium	Malta	Greece
Ireland	Cyprus	Poland	
Lithuania	Croatia	Portugal	
Luxemburg	Denmark	Slovakia	
Romania	Finland	Sweden	
Slovenia	France	Netherlands	
Spain	Germany	Hungary	
	Italy	United Kingdom	

The resulting groups are robust, with the countries that are part of them having approximate values in terms of all recorded values. After analyzing the resulting groups, we observed that life expectancy at birth (years) takes between 74.78 and 78.07 years for group 0 (green), then, Group 1 (blue) records values between 79.03 and 83 years, and for Group 2 (red), we maintained the interval of 78.24–81.79 years. Furthermore, unsatisfactory healthcare registers the highest value in Group 2 (red), with values of 7.3 and 9.1%, respectively. At the same time, the values registered by the other groups are between 0.1 and 2.9% for Group 1 (blue) and 0.1 and 2% for Group 0 (green), respectively. Furthermore, for the three groups, the infant mortality rate is identified as another variable. Group 0 (green) is the one in which the country with the highestvalue of this criterion is observed (Slovenia: 7.06 per 100,000 live births), and at the opposite pole is Cyprus (1.58 per 100,000 of live births) which is part of Group 1 (blue). In the mortality rate (% per month) registered by all groups (0, 1, and 2), the value of 1.56% per month, which is the maximum value was registered by Bulgaria (cluster 0-green) and the minimum value of 0.62% per month, was registered by Italy.

Given both the different priorities of each country on health and the fact that geographical barriers no longer condition the adoption of e-health in terms of technology, which are already overcome in most European countries, we can proceed to the second part of our analysis.

**Figure 5:**
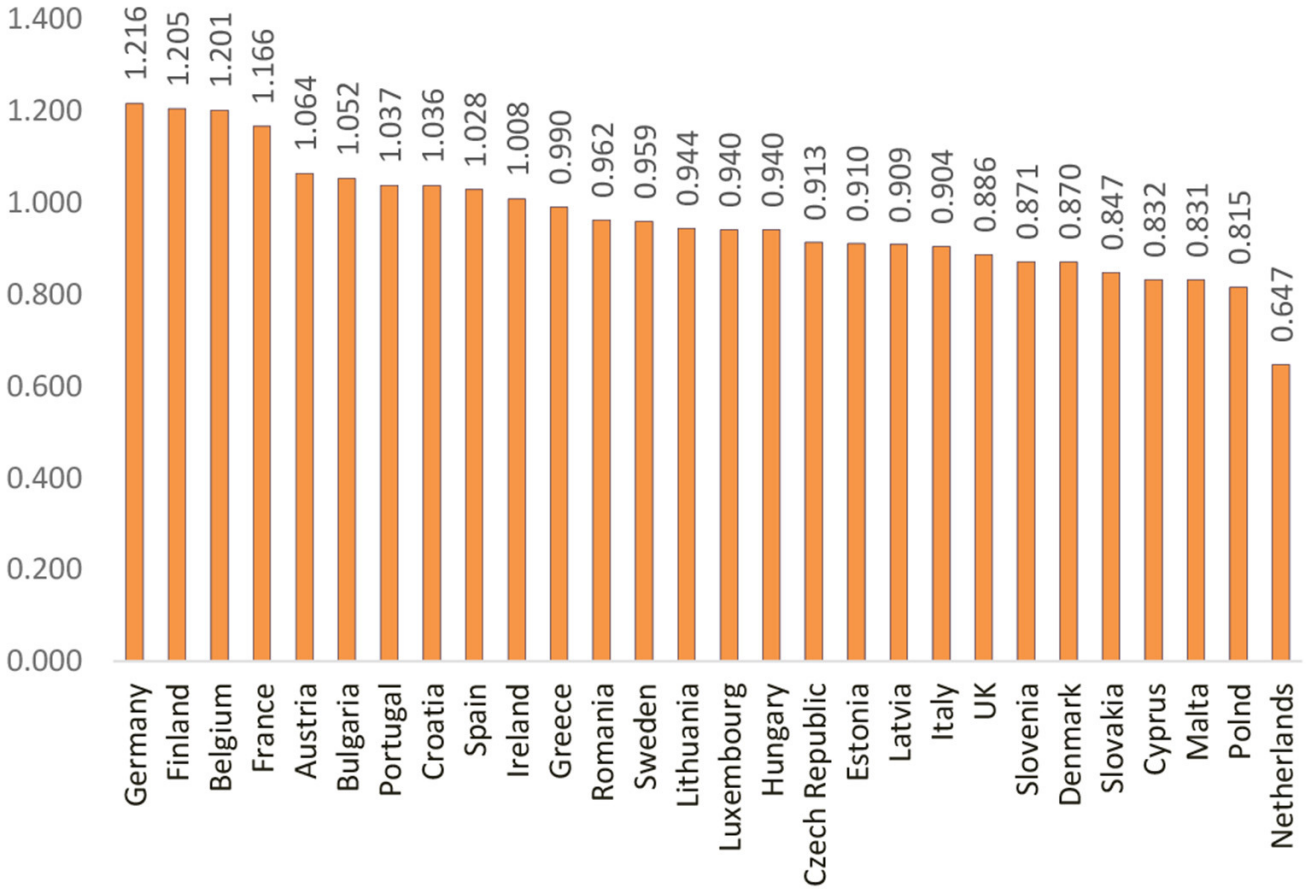
Ranking of the EU countries based on the composite index of disparities in the quality of the public health system.

The next step of the analysis concerns the application of Formula (2) on the criteria that were chosen to assess the public health system and that were taken into account, the calculation of the composite index on the quality of the public health system of European states. Thus, based on the identification of the values of this indicator, European countries are classified as shown in Figure 5.

Figure 5 highlights the countries considered to be the best in terms of the quality of the public health system: Germany, Finland, and Belgium. They gained this position due to the involvement of the government in per capita health spending than other EU countries, offering a wide range of benefits, a high level of service delivery and reasonable access to care, and legislation focusing on long-term care. Instead, we find countries, such as Malta, Poland, and the Netherlands whose indicators of health spending and resources are low due to the main challenges of the health system, namely the long waiting time for services, medical conditions, poor working conditions, and low salaries for medical professionals. At the same time, limited access to health-education, reduced funding for healthcare, and the emigration of medical professionals, call into question, the long-term sustainability and quality of the health system.

Considering the causes underlying the ranking of countries in the top-ranking or at the end of it, we further propose a regression analysis of the composite index of the public health system, as a dependent variable, with the criteria of the public health system, as an independent variable. In this sense, in our models that contain variables related to the public health system, we find the effects of the following criteria: current health expenditure (ccs), health expenditure of the national public administration (csapn), number of dentists (nd), and number of pharmacists (nf), designated based on the R square regression coefficients highlighted in Table 3.

**Table 3:** Coefficients of determination between the composite index public health system and the variables of the quality of the public health system.

**Independent:**	**ccs**	**csapn**	**nps**	**nd**	**nf**
**Coefficients**	0.027^**^	0.029^**^	0.0003^***^	0.002	0.002^***^
**t-Stat**	2.07	2.07	2.72	1.96	3.07
**R-Square**	0.1420	0.1414	0.2217	0.1292	0.2670
***F*****-test**	4.30^**^	4.28^**^	7.40^***^	3.85	9.47^***^

^***^, ^**^ - significant at the level of 1 and 5% respectively.

The results presented in Table 3 indicate that the most significant criterion is represented by nf, immediately followed by number of hospital beds (nps) and by the criteria, ccs and csapn. Based on the study conducted by Lobont et al. (8), in which the results lead to the fact that the number of nurses has a significant role, our study makes a new contribution in the sense of introducing other variables and identifies new criteria, on which the quality of the public health system may depend, such as the number of dentists and pharmacists.

We further explain the problems regarding the quality of the public health system by exemplifying the first and the last ranking countries. Thus, the Netherlands (21.00 per 100,000 inhabitants) faces low values regarding criteria, such as the number of pharmacists or current health expenditure. Simultaneously, despite stable funding and resources, rising healthcare costs (especially long-term care), expensive new technologies, emerging labor shortages, and waiting lists can test the resilience of the health system. The government has addressed many of these issues through reforms and action plans. In contrast, Finland (192.70 per 100,000 inhabitants) has the most significant value in terms of the number of pharmacists due to an appropriate balance between theoretical studies and practical exercises.

Thus, starting from the basic idea that individual health is the result of the quality of the public health system, introducing and evaluating a classification system for the efficiency of the health system will be compared with the composite indices of individual health and the public health system. To carry out the proposed analysis, the relationship between the relative distance from the average related to individual health and the relative distance from the average of a public health system of a person was created.

**Figure 6:**
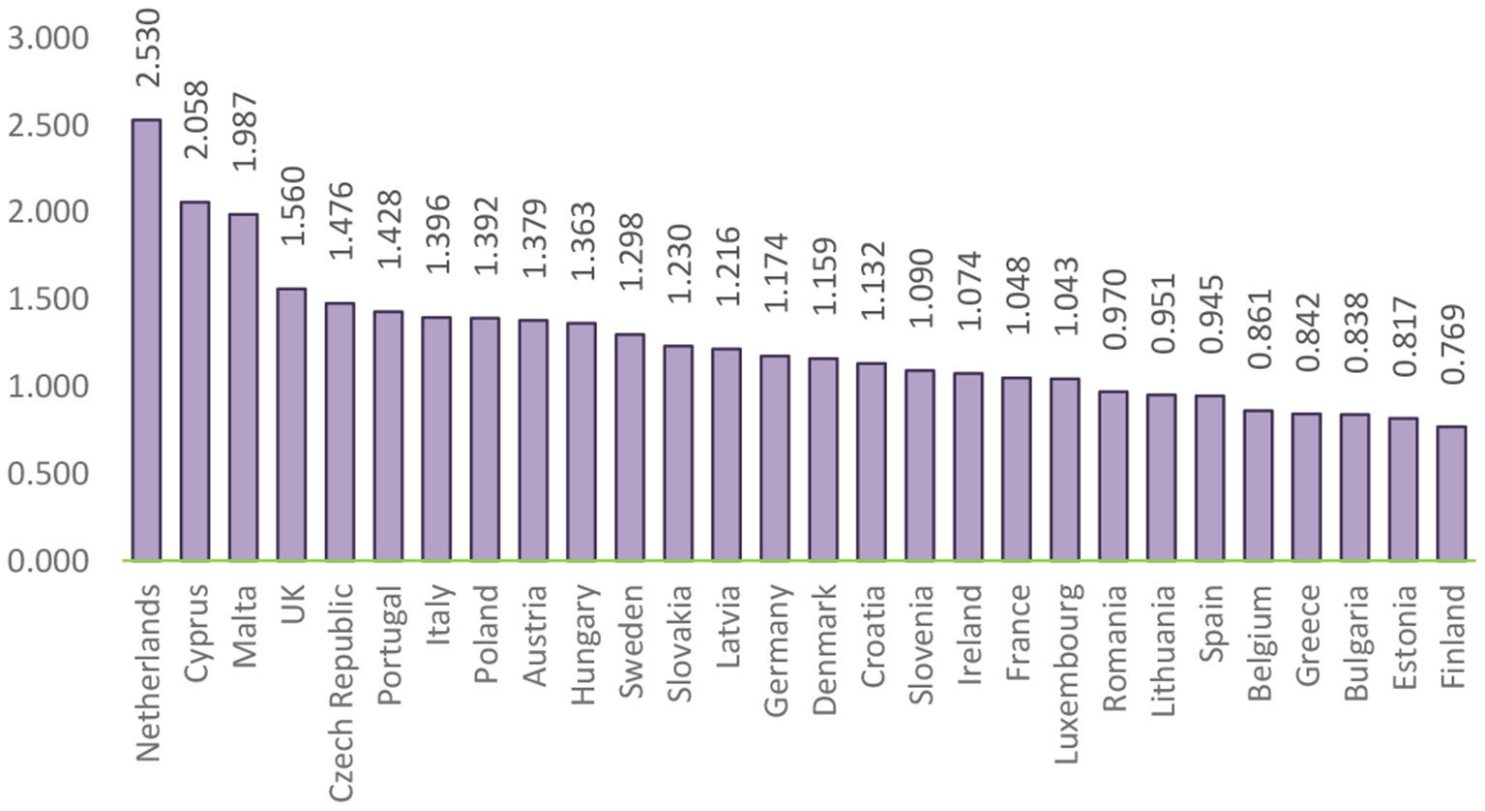
Ranking of the EU countries based on the efficiency of the public health system.

The leaders highlighted in Figure 6 are the Netherlands and Cyprus, closely followed by countries, such as Malta and the United Kingdom, which also have high public health indicators (higher than 1.56). In opposition, we find Bulgaria, Estonia, and Finland, which are at the end of the chart, holding the lowest values in the efficiency indicator of the public health system (<1.84).

The Netherlands records surprising values, being ranked in the first position on analyzing the quality of life index and last in the quality of the public health system. Contrary to these statements, on analyzing some criteria, we can see that the Netherlands has a very long life expectancy (83 years), even above the European average (80 years), which is the highest among the countries analyzed. Although the efficiency of the public health system is high, the Netherlands continues its efforts to support public health priorities and to increase the health promotion and disease prevention activities, i.e., the promotion of healthy lifestyles. At the same time, the position is justified by the measures taken in this country to improve the accountability and governance of the system and to identify the possible cost savings in the health sector administration. Malta is the country with a life expectancy of 82 years, very close to that of the Netherlands.

Moreover, healthcare assessed as unsatisfactory is deficient (both the Netherlands and Malta-0.1%), where both the countries have the lowest values, with a European average of 1.34%. Cyprus also integrates very well into this context, with a life expectancy of 80 years and an equally low reporting of unsatisfactory healthcare (0.1%). Despite the low share of economic resources devoted to healthcare and access problems for some groups of the population considered vulnerable, Cyprus generally enjoy good health than other high-income countries. Indeed, at the base of the specified indicators are some causes that can be considered specific: food, education, and lifestyle considered to be among the healthiest, namely the Mediterranean style, specific to these two countries (Cyprus and Malta). The results of our study are also supported by Jeremic et al. (22), which listed Cyprus as one of the countries with an efficient health system, not only in terms of public spending but also in other external factors, such as the adoption of a healthy Mediterranean diet and a balanced diet.

Bulgaria, Finland, and Estonia are among the countries with the most deficient public health systems, with the quality of life index reflecting low values. The causes could be the significant life expectancy rates (Bulgaria-74 years, Finland-81 years, and Estonia-78 years) and the alarming mortality rate in Bulgaria-1.56% (which is well above the 1.04% average). In terms of the classification of the composite index of the public health system, Finland and Bulgaria occupy important positions at the top of the ranking, with Estonia somewhere in the middle (difference made by health and government expenditures which are lower than in the case of other countries). Although the health system in Finland and Bulgaria is approximately efficient in terms of several indicators, with a relatively high generic penetration of the number of dentists, even pharmacists, and high use of hospital beds, more indicators (expenditure on public health, those of the public administration) suggest the existence of a significant space that can be improved.

## Discussion

Our findings presented in section Results suggest that the efficiency of a public health system can be improved by revising the national health plan, which becomes less of a budgetary instrument and more of a means of planning activities, defining measurable objectives and empowering stakeholders.

In the following section, we discuss the evolution of the composite index of e-health adoption in European countries to observe the changes that took place in 2013–2018 (Table 4).

**Table 4:** Changes in the e-health adoption index between 2013 and 2018.

**Country**	**Composite index e-health adoption 2013**	**Composite index e-health adoption 2018**	**Changes 2013–2018 (+)**
Spain	1.167	2.365	1.198
Estonia	2.133	2.785	0.652
Finland	2.087	2.644	0.557
Sweden	2.010	2.522	0.512
Croatia	1.684	2.180	0.496
UK	2.071	2.517	0.446
Denmark	2.490	2.862	0.372
Lithuania	1.346	1.695	0.349
Latvia	1.497	1.826	0.329
Belgium	1.752	2.067	0.315
Poland	1.540	1.837	0.297
Portugal	1.844	2.118	0.274
Cyprus	1.674	1.934	0.260
Ireland	1.851	2.103	0.252
Slovakia	1.517	1.756	0.239
Bulgaria	1.582	1.809	0.227
Italy	1.972	2.185	0.213
CzechRepublic	1.857	2.063	0.206
Slovenia	1.810	1.998	0.188
Greece	1.605	1.785	0.180
Hungary	1.848	2.028	0.180
France	1.876	2.054	0.178
Malta	1.531	1.695	0.164
Luxembourg	1.614	1.776	0.162
Germany	1.781	1.941	0.160
Austria	1.768	1.914	0.146
Romania	1.695	1.788	0.093

*Own processing after “Benchmarking deployment of e-health among general practitioners” (2013) and “Benchmarking deployment of e-health among general practitioners” (2018)*.

As pointed out in Table 4, the highest increase was found for Spain, namely, the composite index score increased by 1,198 points, from 1,167 in 2013 to 2,365 in 2018. Five Member States had comparable high increases of over 0.4 points: Estonia (an increase of 0.652), Finland (an increase of 0.557 points), Sweden (an increase of 0.512 points), Croatia (an increase of 0.496 points), and the United Kingdom (an increase of 0.446 points). All five Member States with increase of more than 0.4 points are either countries having National Health Service (NHS) or countries in transition.

In contrast, the composite index scores of other Member States increased by <0.2 points: Romania (an increase of 0.093 points), Austria (an increase of 0.146), Germany (an increase of 0.160 points), Luxembourg (an increase of 0.162 points), Malta (an increase of 0.164 points), France (an increase of 0.178 points), Hungary (an increase of 0.180 points), Greece (an increase of 0.180 points), and Slovenia (an increase of 0.188 points). Member States with increases of <0.2 points are a mix of NHS, transition, and social security countries. However, in general, we have seen higher increases among NHS and transition countries than countries with social security.

**Figure 7:**
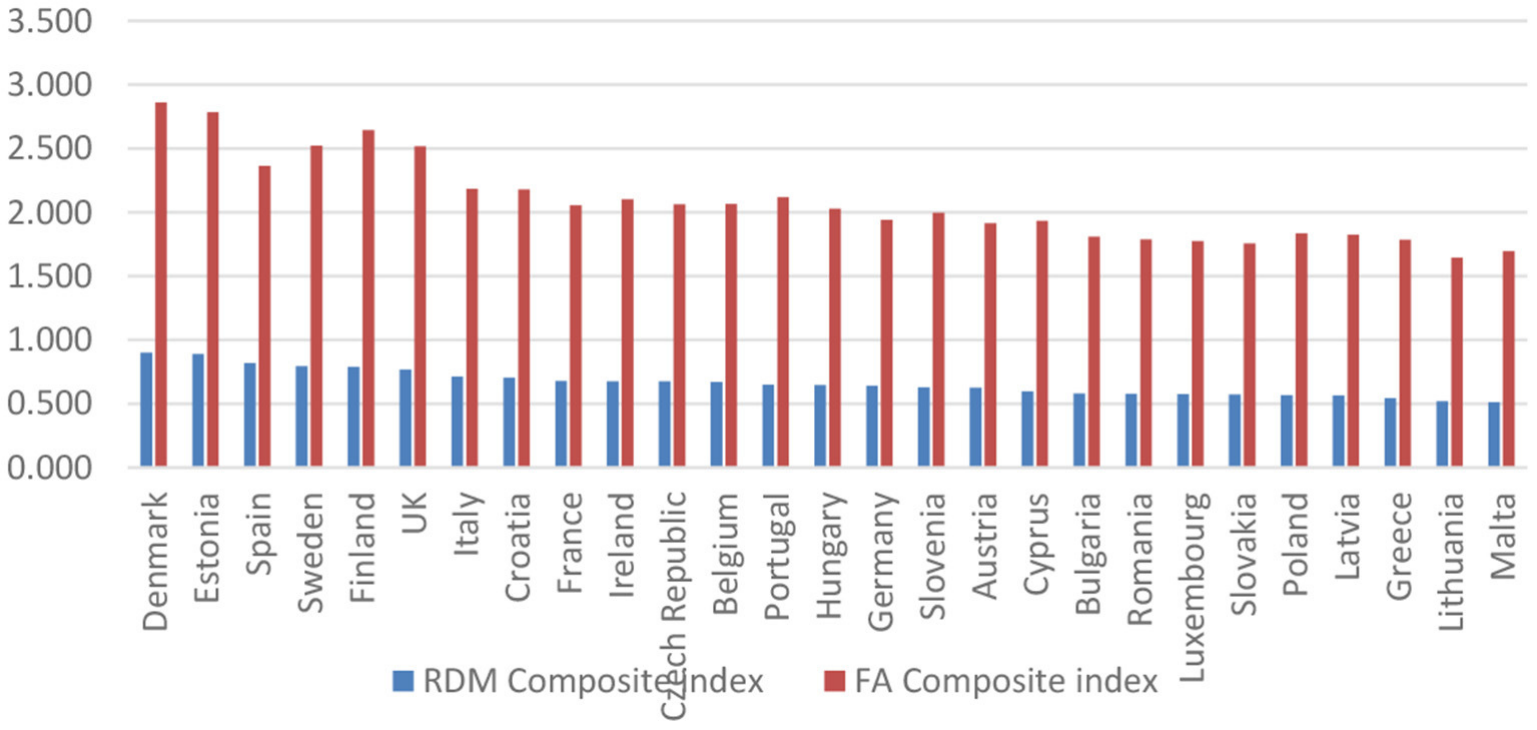
Comparison between the values of RDM and FA composite index.

As robustness of our research, we performed in the following, a comparison between the composite indices for e-health adoption, taken in the first phase from the calculation of the European Commission [by the Factor Analysis (FA) method], and those calculated by the formula (2) (by the relative distances-RDM method), respectively based on the four dimensions that were proposed by the Benchmarking Deployment of eHealth among General Practitioners 2018—Final Report (21). Figure 7 shows the results obtained.

Figure 7 depicts, on the one hand, the countries characterized by a high level of adoption (Denmark, Estonia, Spain, Sweden, Finland, and the United Kingdom), taking into account that e-health is considered routine. On the other hand, the countries with the lowest adoption level (Greece, Lithuania, Luxembourg, Malta, Romania, and Slovakia) suggest that e-health is not widespread.

Furthermore, we revealed the analysis of all the dimensions underlying the formation of the composite index of e-health adoption. Thus, the composite e-health adoption index was based on the four composite indicators described in the previous sections (EHR, HIE, Telemedicine, and PHR).

**Figure 8:**
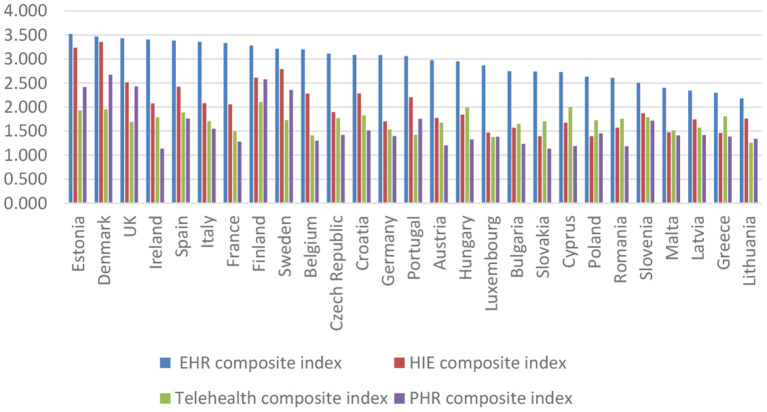
Ranking of the EU countries based on the levels of the composite indicators of e-health adoption.

Figure 8 illustrates the countries that reach a high level in 2018 in terms of the presence and use of e-health adoption dimensions (Estonia, Denmark, UK, Ireland, and Spain), as well as countries with a low level (Lithuania, Greece, Latvia, Malta, and Slovenia). The composite index implies equal weights for each dimension. Therefore, it balances the high adoption of EHR and HIE with the low adoption of telehealth and PHR.

Electronic health record is currently available in all EU countries surveyed. In the majority of the countries, doctors use decision support features (the United Kingdom, Italy, Denmark, Estonia, Spain, Sweden, and Estonia) and administrative data of patients (Luxembourg, Denmark, France, Ireland, and Czech Republic). Adopting HIE is inferior to the adoption of the EHR. The degree of exchange between clinical, administrative, and management data is not yet very high in all the countries analyzed. For example, we observed a significant increase in patient data administration, namely the certification of sick leave and the transfer of prescriptions to pharmacists. An evolution of telehealth can be observed, but it is still characterized by low availability and use in the analyzed countries. Furthermore, training and education features are now available for half of the analyzed countries. The adoption of personal PHR presents a model similar to telehealth. The availability of functionalities regarding the request for appointments and prescriptions have increased, and the functionalities through which patients can view medical records and test results. It can easily be observed that there are some countries (Finland, Denmark, the United Kingdom, Estonia, and Sweden) where these features are more often available than in other countries (Slovenia, Ireland, and Belgium).

## Conclusion

According to our research objective, which aims to identify disparities among the Member States at the European level in e-health adoption, the results reveal the need for significant support for the low level of adoption in countries, such as Malta, Lithuania, Greece, and Latvia. They are also among the countries with a lower quality of life (along with Bulgaria, Finland, and Romania) and a poorly functioning public health system (which can also be witnessed in countries, such as the Netherlands, Poland, Cyprus, and Slovenia). On the other hand, the adoption of e-health is successfully implemented in countries, such as Denmark, Estonia, Spain, Sweden, and Finland, which also have the best level of quality of life and public health system. According to Lobont et al. (8), who conducted a research study on the adoption of e-health, we can say that to a large extent, our results confirm the research hypotheses and are similar to those of this study. After a gap of 3 years, with updated data, no significant changes are observed; therefore, it is essential to adopt measures at the EU level, which should stimulate as much as possible, the implementation of technology in the field of health. Furthermore, the economic and health context that the world faces nowadays highlights the use of ICT in medicine and the importance of digitalization in this sector. The health of the citizens is strongly connected with their level of well-being, along with the performance of the governments in each country.

In 2012, Jeremic et al. analyzed the effectiveness of the public health system and classified the EU countries based on two types of indicators, concerned with the public health systems, and referring to general health. Our results are in contrast to those of this study, in which Greece was ranked first among the EU countries (followed by Belgium and Luxembourg, while Romania, Poland, and Cyprus were at the bottom of the ranking), as a country that invests the most in its health sector, providing the best health services. In terms of the efficiency of health system, the ranking established by Jeremic et al. (22) ranked Cyprus as the most efficient (in our study, Cyprus was placed the second), followed by Ireland, Poland, and Spain, while Latvia, Bulgaria, and Estonia were at the bottom of the health system efficiency ranking. Seke et al. (23) also rated EU countries based on the available data in 2010, in terms of sustainable development and public health. In their case, Norway, Iceland, and UK had a score of approximately 64, Greece was placed sixth (with 61.3), while Hungary, Romania, Lithuania, and Latvia ranked low, with levels lower than 33. Overall, the results obtained in our analysis indicate a significant change in the evolution and development of the public health sector in EU countries.

Looking at the RDM analysis, the countries that directly manage to implement and successfully apply specific e-health functionalities, such as EHR, HIE, telemedicine, and PHR, are Estonia, Denmark, the United Kingdom, Ireland, and Spain, which can be considered to have the highest level of e-health adoption in Europe. Simultaneously, limiting access to health education, low funding for healthcare, and the emigration of medical professionals call into question, the long-term sustainability and quality of the health system in European countries, like Lithuania, Greece, Latvia, Malta, and Slovenia. The countries mentioned above present a low level of implementation and the use of specific dimensions of e-health, such as HIE, Telemedicine, and PHR, which leads to the classification of these countries as the most underperforming countries in e-health adoption. As an alternative to aid, other EU countries (Denmark, Estonia, Finland, and the United Kingdom) can provide some inspiration for the adoption and strengthening of e-health adoption and in the successful implementation of specific dimensions in the medical field (EHR, HIE, Telemedicine, and PHR) in low-adoption countries (Lithuania, Greece, Latvia, Malta, and Slovenia). However, an issue titled “no one fits everyone” and different e-health models probably need to coexist and evolve for each country.

Our result reveals several directions toward which the governing mission of all Member States of the European Union can be directed. The mission of the government mission must be able to create joint visions, objectives, and strategies through which EU Member States can align and reach an agreement. In this case, the creation and existence of national plans must consider the coordination of the Member States toward a common approach that offers the possibility of recovering the economy and digitalization of a country, especially the adoption and implementation of e-health.

The national plans of all Member States must be designed so that they can generate new directions for other states, together creating a framework for the whole of the European Community to follow. The national plan of each Member State must ensure that the other Member States can follow it, essentially that its national plan can generate future benefits for any Member State following it. The EU must provide strategic guidance so that complementarity can be ensured and coordination of these strategic plans is possible.

Another policy measure that is capable of increasing the adoption of e-health is to identify vital national priorities in terms of the health of the citizens. The main priority is to match country specificities in terms of citizens (culture, people education, aversion of people to ICT) with regard to the primary needs of the health system in order to increase the use of e-health dimensions (EHR, HIE, telehealth, and PHR), considering that they have a different degree of adoption and use in the EU Member States.

Taking into account not only the different and complex medical needs of citizens, but also the emergence of crises, as evidenced by the COVID-19 pandemic, each Member State requires the existence of common public health objectives that can regulate interoperability and e-health, more precisely the creation of e-consultation, patient monitoring, and remote care.

The policy measure also highlights the need for each Member State to promote the future benefits and make patients aware of the revolutionary role of technology in the medical system, standardize medical services, and spread the importance of implementing and using information systems, the pillars underpinning e-health adoption.

Moreover, the adoption of e-health in the EU depends on patients and professionals in the field, how to use it in patients, and making distant medical services available to healthcare professionals. As far as medical professionals are concerned, they can be supported by the government by providing funding for innovation, research, and development. New regulations can be developed to increase the adoption of e-health while increasing the level of opportunities and reducing the risks to which patients may be exposed.

Thus, in the context of continued digitization, we affirm the need for public policies, structural reforms, and joint action plans through which EU countries, which alone can reach a significant threshold for all that is essential for the adoption of e-health. Therefore, EU countries need to create common approaches, as evidenced by the uniform and interoperable approach to vaccination, or to develop compelling applications for monitoring and warning with regard to the infection by COVID-19. We note that to help Europe deal with future threats to public health, the EU has proposed a new enhanced EU4 Health program, which will improve support for the health systems of Member States. The EU4 Health aims to contribute to post-COVID-19 recovery, focusing on improving the resilience of health systems and promoting innovation in the health sector.

## Data Availability Statement

The raw data supporting the conclusions of this article will be made available by the authors, without undue reservation.

## Author Contributions

All authors listed have made a substantial, direct and intellectual contribution to the work, and approved it for publication.

## Conflict of Interest

The authors declare that the research was conducted in the absence of any commercial or financial relationships that could be construed as a potential conflict of interest.

## References

[1] Dixon BE. A Roadmap for the Adoption of e-Health. e-Service J. (2007) 5:3–13. 10.2979/esj.2007.5.3.3

[2] Sapirie S. Assessing health information systems. In: Lippeveld T Sauerborn R Bodart C, editors. Design Implementation of Health Information System. Geneva: WHO (2000).

[3] Chaudhry B Wang J Wu S Maglione M Mojica W Roth E. Systematic review: impact of health information technology on quality, efficiency, and costs of medical care. Ann Intern Med. (2006) 144:742–52. 10.7326/0003-4819-144-10-200605160-00125 16702590

[4] Kickbusch I. HealthGovernance: The Health Society. New York, NY: Springer (2007).

[5] Chi Wei S Tiezhu S Shabbir A Nawazish M. Does institutional quality and remittances inflow crowd-in private investment to avoid Dutch Disease? A case for emerging seven (E7) economies. Resour Policy. (2021) 72:102111. 10.1016/j.resourpol.2021.102111

[6] Oh H Rizo C Enkin M Jadad A. What is eHealth? A systematic review of published definitions. World Hosp Health Serv. (2005) 7:32–40. 10.2196/jmir.7.1.e1 15881824

[7] Bloom G Standing H. Future health systems. Why future? Why now?. Soc Sci Med. (2008) 66:2067–75. 10.1016/j.socscimed.2008.01.032 18321628

[8] Lobont OR Vatavu S Brindescu OD Pelin A Chis C. E-health adoption gaps in the decision-making process. Rev Cercetare Interventie Soc. (2019) 65:389–403. 10.33788/rcis.65.24

[9] Popović B Maksimović M. E-health in Bosnia and Herzegovina: exploring the challenges of widespread adoption. In: Badnjevic A. editor. CMBEBIH 2017. IFMBE Proceedings. Singapore: Springer (2017) 62:388–95. 10.1007/978-981-10-4166-2_60

[10] Zayyad MA Toycan M. Factors affecting sustainable adoption of e-health technology in developing countries: an exploratory survey of Nigerian hospitals from the perspective of healthcare professionals. PeerJ. (2018) 6:e4436. 10.7717/peerj.4436 29507830 PMC5835346

[11] Hoque MR Albar A Alam J. Factors influencing physicians' acceptance of e-health in developing country: an empirical study, Int J Health Inform Syst Inform. (2016) 11:58–70. 10.4018/IJHISI.2016010104

[12] Murray E Burns J May C Finch T O'Donnell C Wallace P. Why is it difficult to implement e-health initiatives? A qualitative study. Implement Sci. (2011) 6:6. 10.1186/1748-5908-6-6 21244714 PMC3038974

[13] Devlin AM McGee-Lennon M O'Donnell C Bouamrane MM Agbakoba R O'Connor S. The “Dallas” evaluation team, Delivering digital health and well-being at scale: lessons learned during the implementation of the Dallas program in the United Kingdom, Am Med Inform Assoc. (2016) 23: 48–59. 10.1093/jamia/ocv097 26254480 PMC4713902

[14] Lang A Mertes A. e-Health policy and deployment activities in Europe. Telemed J E Health. (2011) 17:262–8. 10.1089/tmj.2010.0174 21476924

[15] Melchiorre MG Lamura G Barbabella F. eHealth for people with multimorbidity: results from the ICARE4EU project and insights from the “10 e's” by Gunther Eysenbach. PLoS ONE. (2018) 13:e0207292. 10.1371/journal.pone.0207292 30427924 PMC6241125

[16] Peek STM Luijkx KG Vrijhoef HJM Nieboer ME Aarts S van der Voort CS. Understanding changes and stability in the long-term use of technologies by seniors who are aging in place: a dynamical framework. BMC Geriatrics. (2019) 19:236. 10.1186/s12877-019-1241-9 31462214 PMC6712781

[17] Arena C Catuogno S Saggese S Sarto F. The adoption of e-Health in public hospitals. Unfolding the gender dimension of TMT and line managers. Public Manag Rev. (2020) 1–27. 10.1080/14719037.2020.1775280

[18] Johansson A Larsson M Ivarsson B. Patients' experiences with a digital primary health care concept using written dialogues: a pilot study. J Primary Care Commun Health. (2020) 11:215013272091056. 10.1177/2150132720910564 32114868 PMC7052444

[19] Hantrais L Allin P Kritikos M Sogomonjan M Anand PB Livingstone S. Covid-19 and the digital revolution. Contemp Soc Sci. (2021) 16:256–270. 10.1080/21582041.2020.1833234

[20] Codagnone C Lupianez-Villaneuva F. Benchmarking Deployment of eHealth among General Practitioners 2013-Final Report. (2013). Available online at: http://ec.europa.eu/information_society/newsroom/cf/dae/document.cfm?doc_id=4897.

[21] Lupiáñez-Villanueva F Folkvord F Faulíau C. Benchmarking Deployment of eHealth Among General Practitioners 2018-Final Report. (2018). Available online at: https://op.europa.eu/en/publication-detail/-/publication/d1286ce7-5c05-11e9-9c52-01aa75ed71a1/language-en and https://www.rand.org/randeurope/research/projects/benchmarking-ehealth-among-general-practitioners.html.

[22] Jeremić V. Bulajic M Martic M Markovic A Savi,ć G Jeremic D. An evaluation of European countries health systems through distance based analysis. Hippokratia. (2012) 16:170–4. 23935275 PMC3738421

[23] Seke K Petrovic N Jeremic V Vukmirovic J Kilibarda B Martic M. Sustainable development and public health: rating European countries. BMC Public Health. (2013) 13:77, 10.1186/1471-2458-13-77 23356822 PMC3575310

[24] Ceausescu AI. Ierarhizarea jude?elor şi a regiunilor prin cuantificarea inegalitătilor cu ajutorul metodei distantelor relative. In: Analele Univ. “Constatin Brancusi” Tg. Jiu, Seria Economie. Vol. 3, (2011) p. 38–41. Available online at: http://www.utgjiu.ro/revista/ec/pdf/2011-03/5_AURELIAN_IONUT_CEAUSESCU.pdf.

[25] Totan LS Geamanu M Tudose G. Muta?ii Structurale ale For?ei de Muncă din Româniadupă 1990. Romanian Statistical Review, Vol. 9. (2012). Available online at: http://www.revistadestatistica.ro/Articole/2012/RRS09_2012_a5_ro.pdf.

