# Supplementary material for: Challenges on Radical Health Redesign to Reconfigure the Level of e-Health Adoption in EU Countries
Source: Front Public Health. 2021 Jul 20;9:728287. doi: 10.3389/fpubh.2021.728287 (PMC8329364; doi:10.3389/fpubh.2021.728287)
Supplement: Supplementary file 1 [file Table_1.DOCX]

Supplementary Material

Appendix A. Supplementary information of the main variables

**Table A1.** Criteria - Quality of life

| **Nr. crt.** | **Indicator (Criterion)** | **Unit of**  **measure** | **Abbreviation** |  |  | |  |  |  |  |
| --- | --- | --- | --- | --- | --- | --- | --- | --- | --- | --- |
| 1 | Life expectancy at birth | Years | (svn) | |  |  |  |  |  |  |
| 2 | Healthy life years - women | Years | (fvs) | |  |  |  |  |  |  |
| 3 | Healthy years of life - men | Years | (bvs) | |  |  |  |  |  |  |
| 4 | People with long-term illnesses or health problems | (%) | (bps) | |  |  |  |  |  |  |
| 5 | Unsatisfactory medical care | (%) | (amns) | |  |  |  |  |  |  |
| 6 | Infant mortality rate in the country | persons | (mir) | |  |  |  |  |  |  |
| 7 | Mortality rate per month | persons | (rm) | |  |  |  |  |  |  |

**Table A2.** Criteria - Quality of the public health system

| **Nr. crt.** | **Indicator (Criterion)** | **Unit of**  **measure** | **Abbreviation** |  |  | |  |  |  |  |
| --- | --- | --- | --- | --- | --- | --- | --- | --- | --- | --- |
| 1 | Current health expenditures | % of GDP | (ccs) | |  |  |  |  |  |  |
| 2 | Health expenditures of the national  public administration | % of GDP | (csapn) | |  |  |  |  |  |  |
| 3 | Number of hospital beds | per 100.000 inhabitants | (nps) | |  |  |  |  |  |  |
| 4 | Number of dentists | per 100.000 inhabitants | (nd) | |  |  |  |  |  |  |
| 5 | Number of pharmacists | per 100.000 inhabitants | (nf) | |  |  |  |  |  |  |

**Table A3.** EHR overview: sub-dimensions and functionalities

| **sub-dimensions** | **functionalities** |  | | | | | |  |  |  |  |  |
| --- | --- | --- | --- | --- | --- | --- | --- | --- | --- | --- | --- | --- |
| Health Info and Data | symptom  Reason for scheduling  Clinical notes  Vital signs  Treatment results  Medical history  Basic medical parameters  List of problems / diagnoses |  |  |  |  |  |  |  |  |  |  |  |
| Clinical decision support system | Contradictions  Drug interactions  Drug-laboratory interactions  Drug allergy alert  Clinical guidelines and best practices  Warning of a critical laboratory value |  |  |  |  |  |  |  |  |  |  |  |
| Order-entry management | List of drugs  Prescriptions / medicines  immunization  Laboratory test results  Tests ordered |  |  |  |  |  |  |  |  |  |  |  |
| Image | Radiology test images  Radiology test reports |  |  |  |  |  |  |  |  |  |  |  |
| Administrative | Administrative finance / billing  Administrative data on patients |  |  |  |  |  |  |  |  |  |  |  |

**Table A4.** HIE overview: sub-dimensions and functionalities

| **sub-dimensions** | **functionalities** |  | | | | | |  |  |  |  |  |
| --- | --- | --- | --- | --- | --- | --- | --- | --- | --- | --- | --- | --- |
| Clinical data | Exchange of drug lists for patients with other professionals’ / health care providers  Exchange of radiology reports with other  professionals / health care providers  Sharing data on medical patients with other professionals’ / health care providers  Receive and send lab reports and share with other healthcare professionals / providers  Sending / receiving sending and downloading letters  Make appointments with other care providers on behalf of patients  Exchange patient data with any healthcare provider in other countries  Transfer prescriptions to pharmacists |  |  |  |  |  |  |  |  |  |  |  |
| Patient admin | Certify sick leave  Certify disabilities  Patient scheduling requests |  |  |  |  |  |  |  |  |  |  |  |
| Management | Management exchanges administrative data about patients with reimbursements or other care providers  Order cabinet supplies |  |  |  |  |  |  |  |  |  |  |  |

**Table A5.** TeleHealth overview: sub-dimensions and functionalities

| **sub-dimensions** | **functionalities** |  | | | | | |  |  |  |  |  |
| --- | --- | --- | --- | --- | --- | --- | --- | --- | --- | --- | --- | --- |
| Clinical practice | Remote patient monitoring  Consultations with patients |  |  |  |  |  |  |  |  |  |  |  |
| Training | Training / Education  Consultations with other health professionals |  |  |  |  |  |  |  |  |  |  |  |

**Table A6.** Overview of PHR (Personal Health Record): sub-dimensions and functionalities

| **sub-dimensions** | **functionalities** |  | | | | | |  |  |  |  |  |
| --- | --- | --- | --- | --- | --- | --- | --- | --- | --- | --- | --- | --- |
| Clinical information | View medical records  Completion of medical files  View test results |  |  |  |  |  |  |  |  |  |  |  |
| Applications/ Requests | Request recommendations  Request appointments  Request for renewal of prescriptions |  |  |  |  |  |  |  |  |  |  |  |
